# Poly(A)-specific ribonuclease and Nocturnin in squamous cell lung cancer: prognostic value and impact on gene expression

**DOI:** 10.1186/s12943-015-0457-3

**Published:** 2015-11-05

**Authors:** Panagiotis Maragozidis, Eirini Papanastasi, Diana Scutelnic, Athina Totomi, Ioanna Kokkori, Sotirios G. Zarogiannis, Theodora Kerenidi, Konstantinos I. Gourgoulianis, Nikolaos A. A. Balatsos

**Affiliations:** Department of Biochemistry and Biotechnology, University of Thessaly, Ploutonos 26, Larissa, 412 21 Greece; Department of Respiratory Medicine, Faculty of Medicine, University of Thessaly, Biopolis, Larissa, 415 00 Greece; Department of Pneumonology - Oncology, Theagenio Cancer Hospital, Al. Symeonidi 2, Thessaloniki, 540 07 Greece; Department of Physiology, Faculty of Medicine, University of Thessaly, Biopolis, Larissa, 415 00 Greece

**Keywords:** Deadenylases, Prognostic value, Lung cancer, mRNA degradation, Squamous lung carcinoma

## Abstract

**Background:**

Lung cancer is the leading cause of cancer mortality worldwide, mainly due to late diagnosis, poor prognosis and tumor heterogeneity. Thus, the need for biomarkers that will aid classification, treatment and monitoring remains intense and challenging and depends on the better understanding of the tumor pathobiology and underlying mechanisms. The deregulation of gene expression is a hallmark of cancer and a critical parameter is the stability of mRNAs that may lead to increased oncogene and/or decreased tumor suppressor transcript and protein levels. The shortening of mRNA poly(A) tails determines mRNA stability, as it is usually the first step in mRNA degradation, and is catalyzed by deadenylases. Herein, we assess the clinical significance of deadenylases and we study their role on gene expression in squamous cell lung carcinoma (SCC).

**Methods:**

Computational transcriptomic analysis from a publicly available microarray was performed in order to examine the expression of deadenylases in SCC patient samples. Subsequently we employed real-time PCR in clinical samples in order to validate the bioinformatics results regarding the gene expression of deadenylases. Selected deadenylases were silenced in NCI-H520 and Hep2 human cancer cell lines and the effect on gene expression was analyzed with cDNA microarrays.

**Results:**

The *in silico* analysis revealed that the expression of several deadenylases is altered in SCC. Quantitative real-time PCR showed that four deadenylases, PARN, CNOT6, CNOT7 and NOC, are differentially expressed in our SCC clinical samples. PARN overexpression correlated with younger patient age and CNOT6 overexpression with non-metastatic tumors. Kaplan-Meier analysis suggests that increased levels of PARN and NOC correlate with significantly increased survival. Gene expression analysis upon PARN and NOC silencing in lung cancer cells revealed gene expression deregulation that was functionally enriched for gene ontologies related to cell adhesion, cell junction, muscle contraction and metabolism.

**Conclusions:**

Our results highlight the clinical significance of PARN and NOC on the survival in SCC diagnosed patients. We demonstrate that the enzymes are implicated in important phenotypes pertinent to cancer biology and provide information on their role in the regulation of gene expression in SCC. Overall, our results support an emerging role for deadenylases in SCC and contribute to the understanding of their role in cancer biology.

**Electronic supplementary material:**

The online version of this article (doi:10.1186/s12943-015-0457-3) contains supplementary material, which is available to authorized users.

## Background

Lung cancer is the leading cause of cancer mortality worldwide, primarily due to its late diagnosis in advanced stages. Approximately 80 % of lung cancers are classified as non-small-cell lung cancer (NSCLC) and 20 % as small cell lung cancer (SCLC). NSCLC is further distinguished mainly to adenocarcinoma, squamous cell carcinoma (SCC), and other subtypes, with the first two accounting over 80 % of the cases [1, 2]. Over the last decade, encouraging treatments have emerged, particularly for adenocarcinoma. However, this is not the case for SCC as diagnosis is often challenging and several immunohistochemical markers are used for its histological classification [2]. As with all types of cancer, lung cancer is the result of molecular changes occurring in lung tissue cells, leading to the deregulation of several pathways controlling cell growth, differentiation, and cell death [3, 4]. At the molecular level, lung cancer pathogenesis involves mutational, chromosomal and epigenetic changes leading to the activation of oncogenes and/or the inactivation of tumor suppressor genes, reflecting to changes at the protein level [5]. The accumulation of multiple molecular transformations ultimately results in an expression imbalance between oncogenes and tumor suppressor genes that may lead to malignancy [6]. It is evident that there is a need for systematic classification of the molecular counterparts of lung tumors as well as the development of surrogates with respect to their phenotypic components, such as histological tumor type. These will contribute to the development of improved clinical prognostic tools and treatment strategies. In this aspect, much progress has been done over the last years through the molecular profiling of lung cancer using gene array assays for mRNAs and microRNAs. The results of such studies provided molecular signatures that may be useful for the prediction of patient survival and response to therapy [7–10].

Furthermore, the differential expression of cancer-related genes could be the result of perturbations of other mechanisms [11]. Recently it was shown that changes in RNA stability are involved in the pathogenesis of lung cancer and the factors involved could represent new potential targets for its treatment [12]. The regulation of mRNA turnover represents a promising mechanism, since it has been reported that many RNA-degrading enzymes, known as ribonucleases (RNases), are associated with the development of human cancers [13]. The majority of eukaryotic mRNAs are degraded via the deadenylation-dependent pathway, where the shortening of the poly(A) tail is the first and rate-limiting step [14–16]. The reaction is catalyzed by deadenylases, a family of poly(A)-specific ribonucleases. Deadenylases are categorized into two superfamilies, DEDD and EEP. DEDD-type nucleases are named after the conserved catalytic Asp and Glu residues that are dispersed between three exonuclease motifs, which coordinate Mg^2+^ ions, while the members of EEP-type (Exonuclease–Endonuclease–Phosphatase) have conserved catalytic Asp and His residues in their nuclease domains [15]. Recent work has shown that the expression of several deadenylases, including PARN and CNOT7, is altered in acute leukemias, and PARN may represent a promising biomarker and drug target [17, 18]. Furthermore, CNOT6L and PARN deadenylases are linked to the expression of cancer-related mRNAs. CNOT6L, a speculated proto-oncogene product, is linked to the expression of p27Kip1 mRNA, a tumor suppressor which may also function as an oncogene [19–21]. PARN, a major deadenylase in mammalian cells, can potentially act as a tumor suppressor, given that it is activated by the tumor-suppressor BARD1 and it is involved in the degradation of IL-8, VEGF, c-jun, uPA, c-fos and TNF-alpha mRNAs, the levels of which are generally increased in cancers [22–26].

Herein, we used computational transcriptomics and clinical samples to analyze the levels of deadenylases and we found that PARN and NOC elevated expression is related to increased overall survival. Further, we silenced the expression of PARN and NOC and we identified transcripts and gene ontologies (GO) affected by these deadenylases in two cell lines of different cancer origin. These results contribute to our understanding on the role of deadenylases in the stability of mRNAs in SCC and reveal the promising prognostic value of PARN and NOC in this lung cancer subtype.

## Results

### Computational trascriptomic analysis of deadenylases expression in squamous cell lung carcinoma

**Fig. 1:**
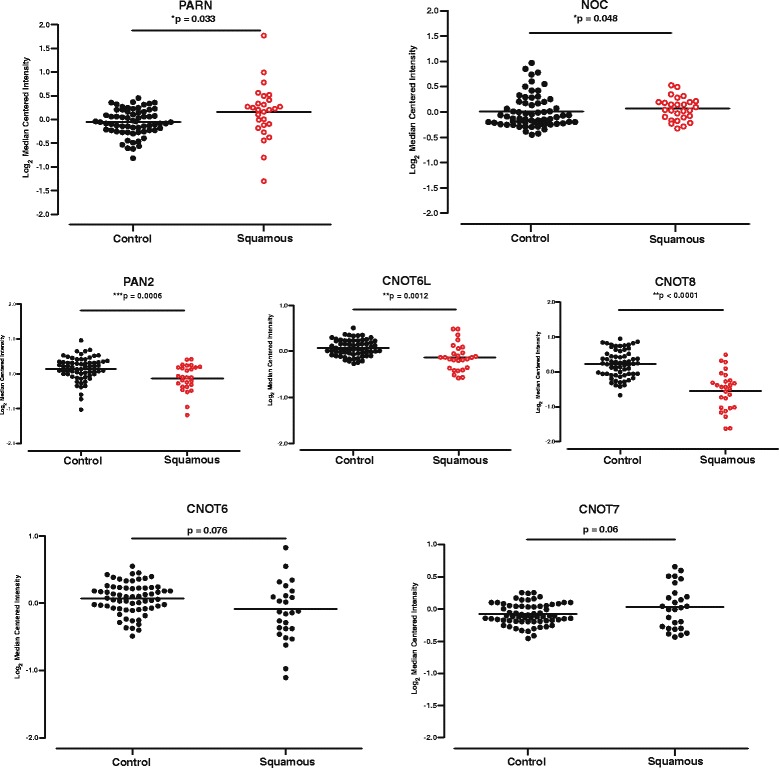
Bioinformatic analysis of expression of deadenylases in Squamous Cell Lung Carcinoma (SCC). Microarray data retrieved from the Oncomine database. PARN (*p* = 0.033) and NOC (*p* = 0.048) are significantly overexpressed, while CNOT6L (*p* = 0.0012), CNOT8 (*p* < 0.0001) and PAN2 (*p* = 0.0006) are significantly underexpressed. The expression of CNOT6 (*p* = 0.076) and CNOT7 (*p* = 0.06) show no-significant alteration in SCC

Based on our previous work on the expression of deadenylases in acute leukemias [18], we opted to assess by means of computational transcriptomics the gene expression profiles of all known deadenylases in SCC as compared to healthy lung tissue serving as control. We therefore used microarray data retrieved from the Hou Lung study included in Oncomine Research Premium Edition Cancer Microarray database (www.oncomine.org), and calculated the differential gene expression levels between normal and SCC tissue [27]. PARN and Nocturnin (NOC), were significantly over-expressed, CNOT6L, CNOT8 and PAN2 were significantly under-expressed, while CNOT6, CNOT7, ANGEL 1 and ANGEL 2 were not differentially expressed between normal controls and SCC (Fig. 1 and Additional file 1: Figure S1). As shown in Fig. 1, the expression of PARN was significantly increased compared to healthy controls (*p* = 0.033), and this was also the case regarding the gene expression of NOC (*p* = 0.048). On the other hand the expressions of CNOT6L (*p* = 0.0012), CNOT8 (*p* < 0.0001) and PAN2 (*p* = 0.0006) were significantly under-expressed in SCC. The gene expressions of CNOT6 (*p* = 0.076), CNOT7 (*p* = 0.06), ANGEL1 (*p* = 0.26) and ANGEL2 (*p* = 0.13) did not differ significantly between the two sample categories (Fig. 1 and Additional file 1: Figure S1).

### Differential expression of deadenylases associates with clinicopathological characteristics and correlates with patient survival

**Fig. 2:**
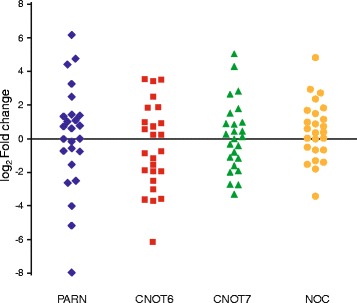
Comparative quantification of deadenylase expression in SCC. PARN (rhombus), CNOT6 (square), CNOT7 (triangle) and NOC (circle) mRNA levels were quantified by real-time PCR in SCC cell extracts and expressed as log_2_ fold-change. Each point corresponds to the relative expression of the deadenylase in malignant tissue compared to the corresponding adjacent non-malignant tissue. The expression of the non malignant tissue was set as 0. *p < 0.05

The computational transcriptomic analysis prompted us to examine the expression of selected deadenylases in samples from patients diagnosed with SCC. We performed comparative quantification in SCC specimens against their matched normal tissues and analyzed the expression levels of deadenylases from both superfamilies, namely DEDD and EEP. Of the examined deadenylases, PARN and CNOT7, belonging to DEDD nucleases, and NOC and CNOT6, belonging to EEP nucleases, were more significantly altered in their expression. PAN2 and CNOT8 were barely detectable in the analyzed clinical samples and were not further investigated. As shown in Fig. 2, in 13 SCC patients (52 %) PARN mRNA levels were elevated [(log_2_ Fold Change (FC) >0)], while in ten patients (40 %) the expression was reduced (log_2_ FC <0). In two patients (8 %) PARN expression was the same between the SCC tissue and the adjacent normal tissue (log_2_ FC = 0). CNOT6 levels were elevated in 12 (48 %) and were reduced in 13 patients (52 %). NOC was overexpressed in 14 patients (56 %), underexpressed in 8 patients (32 %) and in three patients (12 %) showed the same expression profiles. CNOT7 was overexpressed in 12 (48 %) and underexpressed in 12 patients (48 %), while it was unaltered in one patient (4 %).

To associate the results shown in Fig. 2 with clinicopathological characteristics and clinical outcomes, the patients were divided in two groups, where deadenylase expression levels are either increased or decreased, according to the FC difference between the malignant sample and the paired non malignant sample. Patients with no significant alterations were excluded from the analysis (two patients for PARN, one for CNOT7 and three for NOC expression). Such an approach has been previously used for the identification of genes related to RNA metabolism, where patients were divided according to high and low mRNA expression levels [12].

Subsequently, we investigated whether the altered expression of deadenylases is associated with clinical characteristics of SCC patients, namely the age of patients (below *vs* above 65 y.o.), the size of the tumor (T1-2 *vs* T3-4), the lymph node metastasis (N0-1 *vs* N2-3) and distant metastasis (M0 *vs* M1). In Table 1 we summarize the results and show that PARN and CNOT6 are related with some of the clinicopathological characteristics. We observed that PARN is overexpressed in eight out of nine patients under the age of 65 (88.8 %, *p* = 0.03). CNOT6 expression is elevated in 11 patients (78.6 %) with no metastasis, while it was underexpressed in ten patients (91 %, *p* = 0.075) with distant metastasis. This observation suggests that CNOT6 underexpression associates with distant metastasis. We did not observe any significant correlation between CNOT7 and NOC mRNA levels with the examined clinical characteristics.

**Table 1:** Association of PARN, CNOT6, CNOT7 and NOC mRNA levels with clinicopathological characteristics in paired SCC specimens

	Total number of patients	PARN		CNOT6		CNOT7		NOC	
		High^a^ (%)	*p*	High (%)	*p*	High (%)	*p*	High (%)	*p*
Characteristics									
Age									
≤65	9	8 (88.8)	*	7 (77.8)		5 (55.5)		5 (55.5)	
≥65	16	7 (43.8)		6 (37.5)		10 (62.5)		12 (75.0)	
Smoking status									
Smokers	21	11 (52.4)		12 (57.1)		12 (57.1)		14 (66.7)	
Ex-smokers	4	3 (75.0)		1 (25.0)		3 (75.0)		2 (50.0)	
Tumor Size									
T1-T2	9	5 (55.5)		7 (77.8)		4 (45.5)		5 (55.5)	
T3-T4	16	9 (56.3)		6 (37.5)		10 (62.5)		12 (75.0)	
Lymph Node Metastasis									
Negative *(N0-N1)*	6	3 (50)		4 (66.7)		3 (50)		4 (66.7)	
Positive *(N2-N3)*	19	11 (57.9)		9 (47.4)		12 (63.1)		13 (68.4)	
Metastasis									
Negative *(M0)*	14	11 (78.6)		11 (78.6)		10 (71.4)		11 (78.6)	
Positive *(M1)*	11	3 (27.3)		1 (9.0)	**	5 (45.5)		4 (36.4)	

*p < 0.05; **p < 0.01

^a^Samples with increased expression levels

**Fig. 3:**
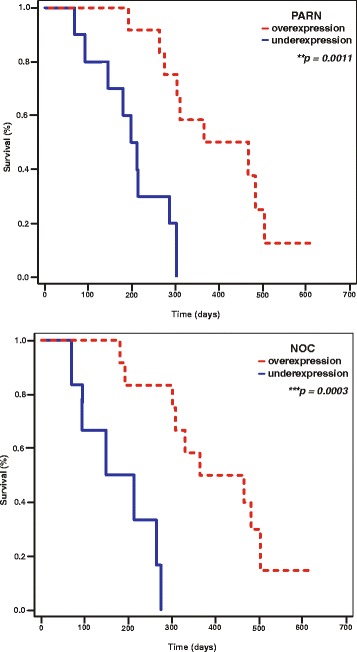
Kaplan-Meier survival curves describe the association between PARN and NOC expression with clinical outcome. Patients’ samples were divided in two groups, where deadenylase expression levels are either increased or reduced, according to the FC difference between the malignant sample and the matched non-pathological one. Overall survival in patients overexpressing PARN, was 7.0 months longer than in patients that underexpress the same enzyme (*p* = 0.0011). For patients overexpressing NOC, overall survival was increased by 7.9 months compared to patients that underexpress the same enzyme (*p* = 0.003)

Next, we generated Kaplan-Meier survival curves to evaluate the significance of PARN, CNOT6, CNOT7 and NOC expression in the prognosis of SCC patients. Our analysis suggests that in patients overexpressing PARN, the overall survival was 7.0 months longer than in patients that underexpress it (median 13.8 months *vs* 6.8 months, *p* = 0.0011) as shown in Fig. 3. For patients overexpressing NOC, overall survival was increased by 7.9 months compared to patients that underexpress the same enzyme (median 13.8 months *vs* 5.9 months, *p* = 0.003) (Fig. 3). We recorded no corresponding survival benefit for patients that overexpress either CNOT6 (median 11.0 months *vs* 9.3 months, *p* = 0.27) or CNOT7 (median 11.3 months *vs* 8.9 months, *p* = 0.0898). These observations suggest that the increased expression of PARN and NOC could be associated with prolonged overall survival in SCC.

### PARN and NOC affect different pathways in squamous lung cancer

The results of the Kaplan-Meier curves prompted us to further investigate the roles of PARN and NOC in tumorigenesis. For this purpose we silenced PARN and NOC expression using specific shRNAs in NCI-H520 cells of SCC origin and examined the impact on gene expression with microarrays. Microarray analysis following PARN silencing identified 322 upregulated (log_2_ FC >2) and 85 downregulated transcripts (log_2_ FC <−2). Additional file 2: Table S1 summarizes the upregulated transcipts; 233 mRNAs and 89 transcripts corresponding to pseudogenes, non-coding RNAs or hypothetical proteins that may represent targets of PARN. To identify the biological processes where the upregulated genes are involved, we performed a functional enrichment analysis of the particular gene set. We used the GeneMANIA online tool (www.genemania.org) and performed a network analysis based on functionally associated data integrated in the software [28]. The analysis revealed the functional enrichment of 36 GOs that are predicted to mediate biological effects as a consequence of the reduced PARN expression (Additional file 3: Table S2). Most of the predicted GOs are associated with cell-cell junction and adhesion, synapse assembly and organization, and various muscle-related processes. On the other hand, NOC silencing in NCI-H520 cells resulted in 307 upregulated and 165 downregulated transcripts. Additional file 4: Table S3 summarizes the upregulated transcripts; 212 mRNAs and 95 transcripts corresponding to pseudogenes, non-coding RNAs, or hypothetical proteins that represent possible targets of NOC. GeneMANIA predicted 118 GOs to be significantly enriched by the gene set of the upregulated genes, and most of them are associated with metabolism-related processes and the circadian rhythm (Additional file 5: Table S4).

**Fig. 4:**
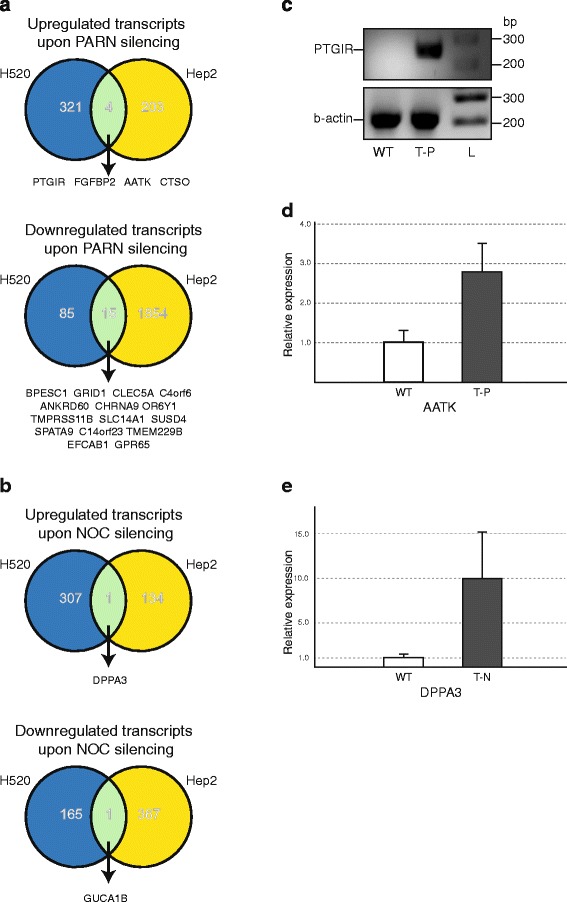
Alterations of gene expression in NCI-H520 and Hep2 cells upon PARN and NOC silencing. **a**, **b** Comparison of gene expression alterations between NCI-H520 and Hep2 cells upon PARN (**a**) and NOC (**b**) silencing. Numbers indicate up- and downregulated transcripts. **c**-**e** Expression of PTGIR, AATK and DPPA3 mRNAs in NCI-H520 cells upon PARN or NOC silencing. The abundance of PTGIR mRNA was determined in wild type (WT) and PARN knock down (T-P) cells by RT-qPCR followed by analysis of the reaction products on 2 % agarose gel and normalized to b-actin mRNA (**c**). The expression levels of AATK (**d**) and DPPA3 (**e**) mRNAs were determined in T-P and NOC knock down (T-N) cells by RT-qPCR and compared to those of WT cells. L, DNA molecular mass marker; numbers on the right indicate DNA base pairs (bp). Error bars represent the standard deviation

To investigate whether these results are SCC-specific we performed the same experiments and subsequent analysis in another cancer cell line, Hep2 cells. Silencing of PARN resulted to the upregulation of 203 transcripts in Hep2, while four transcripts were common in both cell lines (Fig. 4a, Additional file 6: Table S5). Additional file 7: Table S6 summarizes the 20 GOs predicted by GeneMANIA, most of them related to nucleotide metabolism and signaling pathways. From the above common upregulated transcripts PTGIR (Prostaglandin I2 Receptor) and AATK (apoptosis-associated tyrosine kinase) are among the common upregulated transcripts. PTGIR is associated with NSCLC [29] and AATK is associated with other cancer types such as malignant glioma, lung and breast cancers [30, 31]. Among the transcripts that are downregulated upon PARN silencing is C4orf6 (Chromosome 4 Open Reading Frame 6, Expressed In Neuroblastoma), an uncharacterized protein which is associated in neuroblastoma [32].

Also, 1796 transcripts in Hep2 cells were downregulated, with 15 transcripts common in both cell lines (Additional file 6: Table S5), and 20 GOs predicted related to signaling pathways (Additional file 8: Table S7). Furthermore, NOC silencing revealed 134 upregulated transcripts in Hep2 cells, with only one transcript common between the two cell lines; developmental pluripotency associated 3 (*dppa3*). Additionally, 367 transcripts were downregulated in Hep2 cells with one transcript being common between the two cell lines; guanylate cyclase activator 1B (*guca1b*) as shown in Fig. 4b and Additional file 6: Table S5. Also, 8 GOs mainly related to metabolism were predicted using GeneMANIA (Additional file 9: Table S8).

Next, we wished to ascertain that the microarrays results truly reflect changes in the expression of the affected mRNAs upon PARN and NOC knockdown. We performed RT-qPCR on selected transcripts focusing on the upregulated transcripts, as they may represent potential targets of the enzymes, and due to their reported role in lung cancer. Upon PARN silencing, we observed a significant increase of PTGIR mRNA levels (Fig. 4c), while it was undetectable in wild-type cells, in line with previous reports on the limited expression of the receptor in NSCLC [29]. AATK levels were increased more than two fold compared to wild-type cells (Fig. 4d). Following NOC silencing, DPPA3 levels were increased (Fig. 4e).

The previous results suggest that PARN and NOC affected different transcripts and ontologies in SCC cells, and are involved in the regulation of different pathways. Moreover, the silencing of PARN and NOC in two cell lines of different cancer origin, NCI-H520 and Hep2, affected different groups of transcripts with very few genes in common, suggesting a discrete role for each enzyme in different cancer types.

## Discussion

Deadenylases play key role in the regulation of mRNA stability, while several cancer-related genes show either increased or decreased expression. Recent work from our group showed that the levels of expression of several deadenylases -including PARN- are altered in acute leukemias [18]. We, therefore, speculated that deadenylase levels may contribute to the deregulated gene expression in cancer and thus might be of diagnostic and prognostic value. Our previous observation in leukemia led us to examine the expression of deadenylases in lung cancer, since this is the leading cause of cancer mortality worldwide.

As a first step, we interrogated a publicly available microarray dataset in the Oncomine microarray database for deadenylases in SCC. Our *in silico* search showed that the expression of PARN and NOC were significantly increased as compared to healthy controls, while PAN2, CNOT6L and CNOT8 were significantly under-expressed. We therefore examined the expression of these deadenylases using comparative quantification between SCC specimens and their matched non-pathological tissues, and we observed that PARN and CNOT6 overexpression correlated with younger patient age and non-metastatic tumors, respectively. Further, PARN or NOC overexpression is observed in samples from patients with increased overall survival, while in samples derived from non-metastatic tumors the expression of CNOT6 was increased. The median survival of patients that were overexpressing PARN and NOC as compared to those that were underexpressing them, increased by 7.0 and 7.9 months, respectively.

Although none of the deadenylases examined in our study was found exclusively up- or downregulated in SCC, when we grouped the malignant samples in those who overexpress and those who underexpress the same enzyme compared to their matched normal tissue, we observed that 88.8 % of patients <65 y.o. were found to overexpress PARN (*p* = 0.03). Regarding CNOT6, only one out of the 11 patients (<10 %) who had a distant metastasis was overexpressing CNOT6, an observation that could imply a possible protective role of this enzyme over lung cancer invasion and can be a strong indicator of non-metastasis in SCC (*p* = 0.007). Survival analysis using Kaplan-Meier curves showed that patients that overexpressed PARN or NOC had better survival and these genes have the potential to be used in clinical practice.

Our results on *in silico* search in Oncomine database and the biochemical analysis in clinical samples reveal that PARN shows differential expression in SCC. Importantly, PARN overexpression seems to associate with increased overall survival.

The impact of PARN on gene expression have been studied recently. Lee and coworkers presented genome-wide analyses in mouse myoblasts following PARN knockdown, where 40 mRNA were stabilized, including factors required for cell migration and adhesion [33, 34]. Several works aim to elucidate its role in cancer. As mentioned above it has been associated with the expression of cancer-related genes, it is activated by the tumor-suppressor BARD1 and it has been proposed that it could act as a tumor suppressor [13, 35]. In acute leukemias, PARN expression is increased and its phosphorylation status is altered suggesting a potential use as biomarker [18]. In gastric cancer, Zhang and Yan have studied PARN expression in two cell lines of gastric cancer origin, MKN28 and AGS, as well as datasets from Oncomine. They silenced PARN in the cell lines and observed inhibition of proliferation and arrest of cell cycle progression by upregulating p21 mRNA [36]. Although both cell lines were of gastric cacner origin, upon PARN knockdown they showed heterogeneity to p21 mRNA expression suggesting compensatory upregulation of other deadenylases, or a direct stabilization induced by the depletion [36]. However, PARN did affect invasion and showed differential effects on cell motility when compared to the study of Lee and coworkers on mouse myoblasts [33, 36], leading to the suggestion that cancer cells may utilize different regulatory mechanisms of cell motility from mouse myoblasts [36].

In this work, we silenced PARN and NOC in NCI-H520 and Hep2 cancer cells to identify changes in gene expression, possibly related to the altered expression of the enzymes that we observed in SCC samples. Our microarray analysis upon PARN silencing in cells of SCC origin, identified 233 upregulated mRNAs (Additional file 2: Table S1). These may represent direct targets of PARN, as the depletion of the enzyme may result to increased stability of these transcripts. When analyzed for GO functional enrichment, they demonstrated significant enrichment of cell-cell junction and adhesion, synapse assembly and organization, and muscle contraction GOs (Additional file 3: Table S2), in line with previous observations [33]. Additionally, deregulation of cell junction adhesion has been implicated in the process of non-classical, pathological epithelial-mesenchymal transition (EMT), leading to oncogenic transformation and metastasis [37, 38]. These findings could partly justify the results from survival curves, where PARN overexpression correlates with prolonged patient survival. Silencing of PARN in another cancer cell line, Hep2, affected a different population and revealed very few common genes as compared to NCI-H520, suggesting that the effects are SCC-specific (Fig. 4a). Among the transcripts that are downregulated upon PARN silencing is C4orf6 (Chromosome 4 Open Reading Frame 6, Expressed In Neuroblastoma), an uncharacterized protein which is associated in neuroblastoma [32]. The reduced expression of transcripts may imply that these are not direct targets of PARN, and could be attributed to the compensatory activity of another deadenylase, as mentioned above for the modulation of p21 steady-state levels upon PARN silencing in gastric cancer cells [36]. A study on the impact of PARN in genome-wide expression analysis in other types of cancer, such as gastric cancer, would be greatly informative on possible commonly regulated factors/pathways by the enzyme.

Taken together with previous observations in leukemias and gastric cancer these results imply that PARN affects a distinct subset of mRNAs in SCC.

As observed with PARN, NOC overexpression also correlates with prolonged overall survival in SCC (median 13.8 months *vs* 5.9 months, *p* = 0.003) (Fig. 3). Further, NOC silencing in cells of SCC origin resulted in 212 upregulated mRNAs, which might represent targets of the enzyme in SCC. GeneMANIA verifies this rationale; when analyzed for GO functional enrichment, they demonstrated significant enrichment of metabolism-related and circadian rhythm GOs (Additional file 5: Table S4). The comparison of the deregulated transcripts affected by NOC silencing between NCI-H520 and Hep2 cells revealed only two common genes, suggesting that different pathways might be affected in SCC from other cancer types. One of them, DPPA3 (Developmental Pluripotency Associated 3) is also associated with germ cell tumors [39]. NOC is a member of the EEP superfamily of deadenylases that is conserved among eukaryotes and it is widely expressed in many tissues [15, 40]. *NOC* expression is driven by the biological clock, a feature that distinguishes it from other deadenylases. Due to the rhythmic expression, NOC may have cell-specific targets, but mRNAs subject to direct NOC poly(A)-degrading activity *in vivo* have yet to be identified, while it has been proposed that NOC can contribute to the regulation of gene expression independent of its deadenylase activity [40]. Disruption or deregulation of circadian clock is associated with higher cancer incidence, faster progression, and shorter survival [41–45]. Although the association of NOC with pathological conditions is limited so far, single nucleotide polymorphisms (SNPs) within circadian-related genes are considered to confer excess cancer risk or protection from cancer. Thus, the specific core clock genes *Per2* and *Per1* are considered as tumor suppressors linked to tumor numbers and cancer growth rates [46]. Additionally, distorted circadian rhythms have been reported in patients with advanced NSCLC [47] and in a recent work on the association between clock genes and NSCLC in patients, SNP in *NOC* and *Per3* genes demonstrated a significant correlation with genotype and allele frequency, suggesting that polymorphisms might represent a risk factor in NSCLC [48].

Collectively, the microarray and the additional qPCR data indicate potentional targets of PARN and NOC deadenylases. Together with previous studies on the role of PTGIR, AATK and DPPA3 genes in cancer, their transcripts may represent candidate targets to be further examined for their specific role in SCC, and may extend the limited number of the mRNAs that are *bona fide* targets of PARN, including the above-mentioned cancer-related genes, as VEGF, IL-8, u-PA, *c-jun*, *c-fos*, TNF-alpha and p21.

## Conclusions

Our results highlight the association of CNOT6, PARN and NOC expression with clinical data, and the impact of PARN and NOC on gene expression in SCC. Survival seems to be associated to the levels of expression of PARN and NOC, while CNOT6 overexpression may represent a strong indicator of non-metastasis in SCC. The functional genomic analysis upon PARN and NOC depletion in SCC cancer cells, revealed changes in gene expression, which significantly enriched several gene ontologies, mainly associated with cell-cell junction and adhesion, synapse assembly and organization, muscle contraction, metabolism and circadian rhythm. These effects are SCC-specific, as silencing of PARN and NOC in another cancer cell line, affected a different population and revealed very few common genes. As more fundamental knowledge on the biochemical pathways and the biology of lung cancer becomes accessible, there will be novel molecular targets for therapy, better selection of available therapies, as well as more accurate prediction of the patient outcome. Taken together with previous works, our study supports an emerging role of deadenylases in the regulation of gene expression in SCC. Further experimentation is expected to provide more information on the prognostic value of these enzymes in lung cancer.

## Materials and methods

### Computational transcriptomic analysis

We used gene expression data from the Hou Lung cancer study included in the Oncosmine Research Premium Edition Cancer Microarray database (www.oncomine.org) in order to investigate the gene expression profile of deadenylases. The selection of the particular study was performed due to the fact that it was the only one that included surgically excised SCC specimens with healthy lung tissue as control. We analyzed data for PARN, Nocturnin (NOC, Ccrn4l), PAN2, CNOT6 (Ccr4a), CNOT6L, CNOT7 (Caf1), CNOT8, ANGEL1 and ANGEL2 genes in order to assess whether they are differentially expressed in SCC specimens as compared to healthy ones. In order to ensure that the data were generated with the same methodology, we selected gene expression data from a study that included 27 SCC tumor specimens and 65 normal specimens. The gene expression data were log transformed, median centered per array, and the standard deviation was normalized to one per array as described previously [49]. All values from the transformed data were downloaded as Εxcel spreadsheets from Oncomine during July 2013. Gene expression of deadenylases was considered to be over- or under- expressed when its fold-change in the SCC group was significantly different compared to controls (*p* < 0.05). The results were analyzed using GraphPad Prism 4.0 for Mac (GraphPad Software, San Diego, USA). Normal distribution of the data was performed by application of the Kolmogorov-Smirnov normality test. Comparisons of gene expressions were performed with Unpaired *t*-test for parametric data. In cases where variances differed significantly Welch’s correction was applied.

### Human lung tissue specimens

Εndoscopic bronchial biopsy specimens were collected after informed consent from all participants between 2010 and 2012 at the General University Hospital of Larissa, Larissa, Greece and Theagenio Cancer Hospital, Thessaloniki, Greece. Both normal and tumor tissues were collected from the operating room immediately after specimens were removed from patients. The normal tissues were at least 5 cm away from the edge of the corresponding tumors of the matched specimens. In all cases, a pathologist performed histological evaluation of tissue sections. Tumor samples were included in the analysis if the percentage of malignant cells present in the sample were >70 %. Normal lung samples from the same patients were reviewed to confirm that they contained no malignant cells. All samples were divided in two parts: one part was instantly frozen and stored for RNA extraction; the other part was fixed in formalin and embedded in paraffin for routine histological or immunohistochemical tests. Paired tissue samples from 25 patients with SCC were included in the study. Patients’ data, including sex, age at diagnosis, tumor histology, clinical stage, and smoking history, were also collected from their medical records (Table 1). Clinical staging of lung cancers was performed using the revised International System for Staging Lung Cancer [50]. The study was reviewed and approved by the University of Thessaly Ethics Committee.

### RNA extraction and reverse transcription

Total RNA was extracted using the TRI Reagent® Protocol (Sigma). RNA concentration was determined by spectrophotometry (BioPhotometer Plus, eppendorf). One microgram of total RNA was reverse-transcribed by Moloney murine leukemia virus (M-MuLV) reverse transcriptase (PrimeScript RT-PCR kit, Clontech) using oligo(dT) 12–18 as the reverse transcription primer. The reaction mixture was incubated for 50 min at 42 °C and terminated for 15 min at 70 °C.

### Quantitative real-time PCR

Upon RNA extraction, cDNA equivalent to 20 ng of total RNA was subjected to RT-qPCR, in the Mx3005P™ Real-Time PCR System (Stratagene) following the manufacturer’s protocol (KAPA SYBR Fast Universal qPCR kit, KAPA Biosystems). The cycling conditions were as follows: one cycle at 95 °C for 3 min, 40 three-segment cycles (95 °C for 3 s, 60 °C for 30 s and 72 °C for 11 s) and a final dissociation cycle (95 °C for 60 s and progressive rise from 55 to 95 °C). The calculated cycle threshold for the examined transcripts was normalized against the corresponding beta-actin or/and 18S rRNA cycle threshold and expressed as the ratio to the corresponding values. mRNA levels from SCC samples were compared to their paired ones and expressed as log_2_ FC. The PCR and the relative expression results were analyzed using Mx3005P software (Stratagene). The primers were as follows: PTGIR, forward primer 5′-CTTCCAGCGACTCAAGCTCT-3′, reverse primer 5′-CTTCTGCTTTGGACGACGTT-3′ [60 °C annealing temperature (a.t.), 239 bp product size (p.s.)]; FGFBP2, forward primer 5′-CAAGGCCACAGTGAAACTCA-3′, reverse primer 5′-GGCCTTCTTCTTTGCTTCCT-3′ (60 °C a.t., 151 bp p.s.); AATK, forward primer 5′-CCTGGCTCACTGCAAGTACA-3′, reverse primer 5′-GCTCAAAGAGCTCCCAGATG-3′ (60 °C a.t., 179 bp p.s.); DPPA3, forward primer 5′-CACAAATGCTCACCGAAGAA-3′, reverse primer 5′-TTCGATTTCCCTGAGGACTG-3′ (60 °C a.t., 182 bp p.s.). Primer sequences for deadenylases used as previously described [18].

### Cell culture and silencing of deadenylases

NCI-H520 cells originate from human squamous cell carcinoma [51] and are widely used in lung cancer studies. Hep2 (or HEp-2) cell line was originally described to derive from epidermoid carcinoma of the larynx [52]. Although suggested as a HeLa cross-contaminant [53], Hep2 is used in studies considered of laryngeal origin (for instance [54, 55]). NCI-H520 and Hep2 cell lines were routinely maintained in MEM and RPMI-1640 respectively (Biosera), supplemented with 10 % fetal bovine serum (FBS, Biosera) in a humidified atmosphere of 5 % CO_2_ at 37 °C.

NCI-H520 and Hep2 cells grown into 6-well plates were transfected with 6 μg plasmid DNA containing shRNAs sequences designed to silence the selected deadenylase for 72 h, or shRNA with no homology to known gene sequences as negative control, using cationic liposomes (Xfect, Clontech) according to manufacturer’s instructions. The following shDNA plasmids were used to silence the respective deadenylases (MISSION® shRNA, SIGMA): PARN (NM_002582), NOCTURNIN (NM_012118), and non-targeting control (SHC016). Puromycin selection (6 μg/mL) was performed 12 h after transfection according to the manufacturer.

### DNA microarrays and gene ontology prediction

Gene expression analysis of NCI-H520 and Hep2 cells upon deadenylase silencing was performed at NIMGenetics (Madrid, Spain) by using SurePrint G3 Human GE 60 K Microarray and Whole Human Genome Oligo Microarrays 44 k, respectively (Agilent Technologies, Inc., Santa Clara, CA). Initial analysis of the microarray data was performed with cut-off fold-change value ± 2. To exclude any possible transcripts affected by the transfection procedure itself, we used non-Τarget shRNA (Sigma), pLKO.1 empty vector (Sigma) and wild type cells as controls of expression. The microarray data have been deposited in the Gene Expression Omnibus database (www.ncbi.nlm.nih.gov/geo, accession numbers GSE67536 and GSE67598). The upregulated genes arising from the deadenylases silencing were used to predict significantly enriched gene ontologies (GO). GO analysis was performed using GeneMANIA (www.genemania.org) on February 1st 2015 [28].

### Generation of Venn diagrams

Venn diagrams were generated using the Venny software (http://bioinfogp.cnb.csic.es/tools/venny). The genes that were found to be more than 2-fold over- or under- expressed in the microarrays that were performed in the two cells lines (NCI-H520 and Hep2) where PARN and NOC were silenced, were tabulated in lists (Additional file 2: Table S1 and Additional file 4: Table S3). These lists were matched by cell type and by gene over- and underexpression status and were included in Venny generating the Venn diagrams shown in Fig. 4a and b.

## Additional files

Additional file 1: Figure S1. Bioinformatic analysis of expression of ANGEL 1 and ANGEL 2 in SCC. Microarray dataretrieved from the Oncomine database. ANGEL1 (p = 0.26) and ANGEL2 (p = 0.13). (PDF 276 kb) Additional file 2: Table S1. Upregulated transcripts upon PARN silencing in H520 cells. (XLSX 34 kb) Additional file 3: Table S2. Functional Enrichment Analysis of genes with differentially increased expression after PARN silencing in NCI-H520 cells. (DOCX 14 kb) Additional file 4: Table S3. Upregulated transcripts upon NOC silencing in H520 cells. (XLSX 33 kb) Additional file 5: Table S4. Functional Enrichment Analysis of genes with differentially increased expression after NOC silencing in NCI-H520 cells. (DOCX 19 kb) Additional file 6: Table S5. Common up- and downregulated transcripts after PARN or NOC silencing in NCI-H520 and Hep2 cells. (DOCX 13 kb) Additional file 7: Table S6. Functional Enrichment Analysis of genes with differentially increased expression after PARN silencing in both NCI-H520 and Hep2 cells. (DOCX 12 kb) Additional file 8: Table S7. Functional Enrichment Analysis of genes with differentially reduced expression after PARN silencing in both NCI-H520 and Hep2 cells. (DOCX 12 kb) Additional file 9: Table S8. Functional Enrichment Analysis of genes with differentially reduced expression after NOC silencing in both NCI-H520 and Hep2 cells. (DOCX 11 kb)

## Abbreviations

SCC: Squamous cell lung carcinoma
SCLC: Small cell lung cancer
NSCLC: Non-small-cell lung cancer
RNases: Ribonucleases
EEP: Exonuclease–endonuclease–phosphatase
FC: Fold-change
PARN: Poly(A)-specific ribonuclease
NOC: Nocturnin
GO: Gene ontology
