# Supplementary figures and images for: Poly(A)-specific ribonuclease and Nocturnin in squamous cell lung cancer: prognostic value and impact on gene expression
Source: Mol Cancer. 2015 Nov 5;14:187. doi: 10.1186/s12943-015-0457-3 (PMC4635609; doi:10.1186/s12943-015-0457-3)

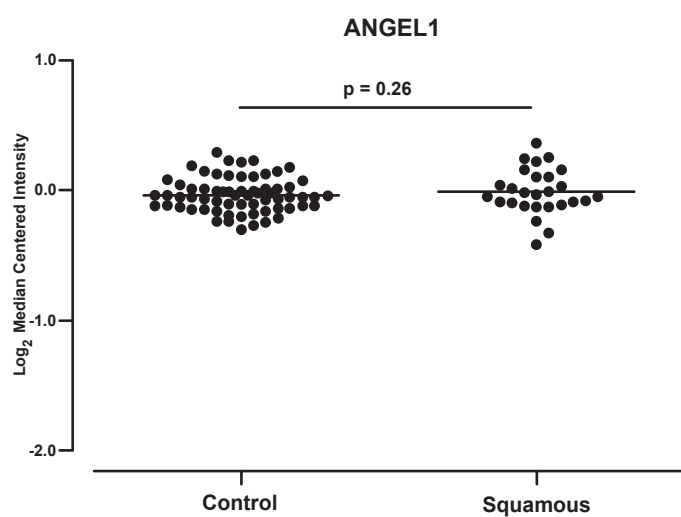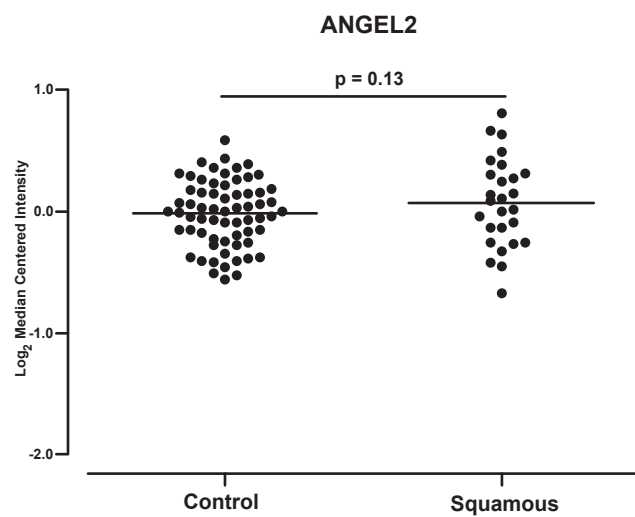

Supplement: Additional file 1: Figure S1. — Bioinformatic analysis of expression of ANGEL 1 and ANGEL 2 in SCC. Microarray dataretrieved from the Oncomine database. ANGEL1 (p = 0.26) and ANGEL2 (p = 0.13). (PDF 276 kb) [file 12943_2015_457_MOESM1_ESM.pdf]
