# Supplementary material for: Poly(A)-specific ribonuclease and Nocturnin in squamous cell lung cancer: prognostic value and impact on gene expression
Source: Mol Cancer. 2015 Nov 5;14:187. doi: 10.1186/s12943-015-0457-3 (PMC4635609; doi:10.1186/s12943-015-0457-3)
Supplement: Additional file 3: Table S2. — Functional Enrichment Analysis of genes with differentially increased expression after PARN silencing in NCI-H520 cells. (DOCX 14 kb) [file 12943_2015_457_MOESM3_ESM.docx]

**Additional file 3: Table S2.** Functional Enrichment Analysis of genes with differentially increased expression after PARN silencing in NCI-H520 cells.

| **Function** | **FDR** | **Coverage** |
| --- | --- | --- |
| **query genes** | **n/a** | **233 / 233** |
| *calcium-dependent cell-cell adhesion* | 1.17E-12 | 12 / 20 |
| *cell junction assembly* | 5.36E-9 | 21 / 164 |
| *cell junction organization* | 5.36E-9 | 22 / 181 |
| *adherens junction organization* | 2.51E-6 | 13 / 76 |
| *cell-cell adhesion* | 1.45E-5 | 21 / 256 |
| *cell-cell junction organization* | 2.22E-5 | 16 / 150 |
| *blood microparticle* | 1.18E-4 | 13 / 108 |
| *synapse assembly* | 7.63E-4 | 9 / 54 |
| *hemidesmosome assembly* | 2.24E-3 | 5 / 12 |
| *platelet degranulation* | 2.46E-3 | 10 / 82 |
| *synapse organization* | 2.46E-3 | 10 / 82 |
| *cardiac muscle tissue morphogenesis* | 4.58E-3 | 7 / 38 |
| *muscle filament sliding* | 4.58E-3 | 7 / 38 |
| *actin-myosin filament sliding* | 4.58E-3 | 7 / 38 |
| *muscle tissue morphogenesis* | 5.74E-3 | 7 / 40 |
| *muscle organ morphogenesis* | 5.74E-3 | 7 / 40 |
| *platelet alpha granule* | 1.06E-2 | 8 / 61 |
| *actin-mediated cell contraction* | 1.55E-2 | 7 / 47 |
| *platelet alpha granule lumen* | 1.7E-2 | 7 / 48 |
| *muscle system process* | 1.76E-2 | 14 / 211 |
| *muscle contraction* | 2.15E-2 | 13 / 188 |
| *I band* | 3.23E-2 | 7 / 54 |
| *myofibril* | 4.95E-2 | 9 / 102 |
| *structural constituent of muscle* | 4.95E-2 | 6 / 41 |
| *actin filament-based movement* | 4.95E-2 | 7 / 59 |
| *contractile fiber part* | 4.95E-2 | 9 / 102 |
| *secretory granule lumen* | 6.5E-2 | 7 / 62 |
| *exocytosis* | 7.2E-2 | 12 / 191 |
| *cardiac muscle tissue development* | 7.2E-2 | 8 / 85 |
| *ventricular cardiac muscle tissue morphogenesis* | 7.2E-2 | 5 / 29 |
| *ventricular cardiac muscle tissue development* | 7.2E-2 | 5 / 29 |
|  |  |  |
| *Z disc* | 7.2E-2 | 6 / 45 |
| *sarcomere* | 7.2E-2 | 8 / 86 |
| *contractile fiber* | 7.75E-2 | 9 / 112 |
| *striated muscle tissue development* | 9.7E-2 | 10 / 142 |
| *cell-substrate junction assembly* | 9.91E-2 | 6 / 49 |

*FDR: false discovery rate
