# Supplementary material for: Poly(A)-specific ribonuclease and Nocturnin in squamous cell lung cancer: prognostic value and impact on gene expression
Source: Mol Cancer. 2015 Nov 5;14:187. doi: 10.1186/s12943-015-0457-3 (PMC4635609; doi:10.1186/s12943-015-0457-3)
Supplement: Additional file 5: Table S4. — Functional Enrichment Analysis of genes with differentially increased expression after NOC silencing in NCI-H520 cells. (DOCX 19 kb) [file 12943_2015_457_MOESM5_ESM.docx]

**Additional file 5: Table S4.** Functional Enrichment Analysis of genes with differentially increased expression after NOC silencing in NCI-H520 cells.

| **Function** | **FDR** | **Coverage** |
| --- | --- | --- |
| **query genes** | **n/a** | **212 / 212** |
| *triglyceride-rich lipoprotein particle remodeling* | 8.26E-7 | 7 / 11 |
| *very-low-density lipoprotein particle remodeling* | 8.26E-7 | 7 / 11 |
| *triglyceride-rich lipoprotein particle* | 7.62E-6 | 7 / 15 |
| *very-low-density lipoprotein particle* | 7.62E-6 | 7 / 15 |
| *reverse cholesterol transport* | 1.07E-5 | 7 / 16 |
| *protein activation cascade* | 1.11E-5 | 11 / 61 |
| *high-density lipoprotein particle* | 1.28E-5 | 7 / 17 |
| *cholesterol homeostasis* | 1.34E-5 | 10 / 50 |
| *sterol homeostasis* | 1.34E-5 | 10 / 50 |
| *plasma lipoprotein particle organization* | 2.68E-5 | 8 / 29 |
| *protein-lipid complex subunit organization* | 3.27E-5 | 8 / 30 |
| *plasma lipoprotein particle* | 5.46E-5 | 7 / 22 |
| *plasma lipoprotein particle assembly* | 5.46E-5 | 6 / 14 |
| *detection of stimulus* | 5.46E-5 | 18 / 230 |
| *plasma lipoprotein particle remodeling* | 5.46E-5 | 7 / 23 |
| *protein-lipid complex remodeling* | 5.46E-5 | 7 / 23 |
| *macromolecular complex remodeling* | 5.46E-5 | 7 / 23 |
| *protein-lipid complex* | 5.46E-5 | 7 / 23 |
| *cholesterol efflux* | 5.46E-5 | 8 / 33 |
| *protein-lipid complex assembly* | 7.37E-5 | 6 / 15 |
| *lipid homeostasis* | 1.67E-4 | 10 / 70 |
| *humoral immune response mediated by circulating immunoglobulin* | 2.24E-4 | 5 / 10 |
| *sterol transporter activity* | 3.87E-4 | 5 / 11 |
| *detection of abiotic stimulus* | 5.64E-4 | 12 / 122 |
| *phospholipid transport* | 9.64E-4 | 7 / 35 |
| *blood microparticle* | 1.07E-3 | 11 / 108 |
| *alcohol binding* | 1.08E-3 | 8 / 51 |
| *high-density lipoprotein particle remodeling* | 1.32E-3 | 5 / 14 |
| *regulation of plasma lipoprotein particle levels* | 1.4E-3 | 7 / 38 |
| *muscle filament sliding* | 1.4E-3 | 7 / 38 |
| *actin-myosin filament sliding* | 1.4E-3 | 7 / 38 |
| *cholesterol transport* | 1.6E-3 | 8 / 55 |
| *sterol transport* | 1.6E-3 | 8 / 55 |
| *lipid localization* | 1.92E-3 | 11 / 118 |
| *detection of external stimulus* | 1.92E-3 | 11 / 118 |
| *quaternary ammonium group binding* | 2.19E-3 | 5 / 16 |
| *actin filament-based movement* | 2.45E-3 | 8 / 59 |
| *lipoprotein particle receptor binding* | 2.82E-3 | 5 / 17 |
| *positive regulation of steroid metabolic process* | 2.82E-3 | 5 / 17 |
| *actin-mediated cell contraction* | 4.77E-3 | 7 / 47 |
| *regulation of triglyceride metabolic process* | 4.91E-3 | 5 / 19 |
| *phototransduction, visible light* | 5.34E-3 | 9 / 87 |
| *regulation of lipid catabolic process* | 5.8E-3 | 6 / 33 |
| *cholesterol transporter activity* | 5.8E-3 | 4 / 10 |
| *cholesterol metabolic process* | 6.6E-3 | 8 / 69 |
| *blood coagulation, fibrin clot formation* | 7.46E-3 | 5 / 21 |
| *organophosphate ester transport* | 8.06E-3 | 7 / 52 |
| *detection of visible light* | 8.78E-3 | 9 / 94 |
| *immunoglobulin mediated immune response* | 8.79E-3 | 6 / 36 |
| *sequence-specific DNA binding RNA polymerase II transcription factor activity* | 8.91E-3 | 13 / 199 |
| *phototransduction* | 1.05E-2 | 9 / 97 |
| *lipid transport* | 1.05E-2 | 11 / 147 |
| *steroid esterification* | 1.06E-2 | 4 / 12 |
| *sterol esterification* | 1.06E-2 | 4 / 12 |
| *cholesterol esterification* | 1.06E-2 | 4 / 12 |
| *B cell mediated immunity* | 1.06E-2 | 6 / 38 |
| *neutral lipid catabolic process* | 1.19E-2 | 5 / 24 |
| *acylglycerol catabolic process* | 1.19E-2 | 5 / 24 |
| *organic hydroxy compound transport* | 1.27E-2 | 9 / 101 |
| *muscle system process* | 1.35E-2 | 13 / 211 |
| *detection of light stimulus* | 1.35E-2 | 9 / 102 |
| *sterol metabolic process* | 1.42E-2 | 8 / 80 |
| *muscle contraction* | 1.82E-2 | 12 / 188 |
| *regulation of sterol transport* | 1.91E-2 | 5 / 27 |
| *complement activation* | 1.91E-2 | 6 / 43 |
| *regulation of cholesterol transport* | 1.91E-2 | 5 / 27 |
| *plasma lipoprotein particle clearance* | 2.26E-2 | 5 / 28 |
| *lipid transporter activity* | 2.34E-2 | 6 / 45 |
| *endoplasmic reticulum lumen* | 2.71E-2 | 10 / 140 |
| *skeletal muscle contraction* | 2.85E-2 | 4 / 16 |
| *positive regulation of lipid catabolic process* | 2.85E-2 | 4 / 16 |
| *adenylate cyclase-modulating G-protein coupled receptor signaling pathway* | 2.85E-2 | 8 / 90 |
| *cholesterol binding* | 2.92E-2 | 5 / 30 |
| *rhythmic process* | 3.26E-2 | 7 / 69 |
| *retinoid metabolic process* | 3.53E-2 | 7 / 70 |
| *glycerolipid catabolic process* | 3.86E-2 | 5 / 32 |
| *diterpenoid metabolic process* | 4.11E-2 | 7 / 72 |
| *response to light stimulus* | 4.21E-2 | 12 / 210 |
| *G-protein coupled receptor signaling pathway, coupled to cyclic nucleotide second messenger* | 4.88E-2 | 9 / 125 |
| *sterol binding* | 4.88E-2 | 5 / 34 |
| *endocrine pancreas development* | 4.88E-2 | 5 / 34 |
| *regulation of transmembrane transport* | 5.03E-2 | 9 / 126 |
| *peptidyl-glutamic acid modification* | 5.03E-2 | 4 / 19 |
| *steroid binding* | 5.32E-2 | 6 / 54 |
| *regulation of lipid transport* | 5.82E-2 | 6 / 55 |
| *terpenoid metabolic process* | 5.94E-2 | 7 / 78 |
| *positive regulation of lipid metabolic process* | 5.94E-2 | 7 / 78 |
| *regulation of fatty acid biosynthetic process* | 5.94E-2 | 4 / 20 |
| *protein heterodimerization activity* | 6.12E-2 | 11 / 190 |
| *regulation of lipid biosynthetic process* | 6.21E-2 | 7 / 79 |
| *triglyceride metabolic process* | 6.21E-2 | 7 / 79 |
| *actomyosin structure organization* | 6.47E-2 | 5 / 37 |
| *sensory perception of chemical stimulus* | 6.55E-2 | 7 / 80 |
| *regulation of lipid metabolic process* | 6.55E-2 | 10 / 162 |
| *steroid metabolic process* | 7.48E-2 | 11 / 196 |
| *regulation of glutamate receptor signaling pathway* | 7.5E-2 | 4 / 22 |
| *regulation of Cdc42 protein signal transduction* | 7.5E-2 | 4 / 22 |
| *triglyceride homeostasis* | 7.5E-2 | 4 / 22 |
| *triglyceride catabolic process* | 7.5E-2 | 4 / 22 |
| *ionotropic glutamate receptor complex* | 7.5E-2 | 4 / 22 |
| *glycoprotein binding* | 7.5E-2 | 5 / 39 |
| *acylglycerol homeostasis* | 7.5E-2 | 4 / 22 |
| *phospholipid binding* | 7.72E-2 | 11 / 199 |
| *detection of chemical stimulus involved in sensory perception* | 7.72E-2 | 6 / 60 |
| *negative regulation of cell differentiation* | 7.72E-2 | 13 / 266 |
| *transcription regulatory region DNA binding* | 7.72E-2 | 13 / 266 |
| *regulatory region DNA binding* | 7.85E-2 | 13 / 267 |
| *regulatory region nucleic acid binding* | 7.85E-2 | 13 / 267 |
| *acylglycerol metabolic process* | 8.01E-2 | 7 / 85 |
| *neutral lipid metabolic process* | 8.01E-2 | 7 / 85 |
| *negative regulation of cholesterol transport* | 8.01E-2 | 3 / 10 |
| *negative regulation of sterol transport* | 8.01E-2 | 3 / 10 |
| *sensory perception* | 8.11E-2 | 12 / 235 |
| *sensory perception of smell* | 8.43E-2 | 5 / 41 |
| *structural constituent of muscle* | 8.43E-2 | 5 / 41 |
| *endocrine system development* | 8.98E-2 | 6 / 63 |
| *circadian rhythm* | 9.29E-2 | 5 / 42 |
| *lipoprotein metabolic process* | 9.93E-2 | 7 / 89 |
