# Supplementary material for: Poly(A)-specific ribonuclease and Nocturnin in squamous cell lung cancer: prognostic value and impact on gene expression
Source: Mol Cancer. 2015 Nov 5;14:187. doi: 10.1186/s12943-015-0457-3 (PMC4635609; doi:10.1186/s12943-015-0457-3)
Supplement: Additional file 6: Table S5. — Common up- and downregulated transcripts after PARN or NOC silencing in NCI-H520 and Hep2 cells. (DOCX 13 kb) [file 12943_2015_457_MOESM6_ESM.docx]

**Additional file 6: Table S5.** Common up- and downregulated transcripts after PARN or NOC silencing in NCI-H520 and Hep2 cells.

| *Common upregulated transcripts after PARN silencing* | |
| --- | --- |
| **PTGIR** | Prostaglandin I2 Receptor |
| **FGFBP2** | Fibroblast Growth Factor Binding Protein |
| **AATK** | apoptosis-associated tyrosine kinase |
| **CTSO** | Cathepsin O |
|  | |
| *Common downregulated transcripts after PARN silencing* | |
| **BPESC1** | Blepharophimosis, Epicanthus Inversus And Ptosis, Candidate 1  (Non-Protein Coding) |
| **GRID1** | Glutamate Receptor, Ionotropic, Delta 1 |
| **CLEC5A** | C-Type Lectin Domain Family 5, Member A |
| **C4orf6** | Chromosome 4 Open Reading Frame 6, Expressed In Neuroblastoma, Uncharacterized Protein C4orf6 |
| **ANKRD60** | Ankyrin Repeat Domain 60 |
| **CHRNA9** | Cholinergic Receptor, Nicotinic, Alpha 9 (Neuronal) |
| **OR6Y1** | Olfactory Receptor, Family 6, Subfamily Y, Member 1 |
| **TMPRSS11B** | Transmembrane Protease, Serine 11B |
| **SLC14A1** | Solute Carrier Family 14 (Urea Transporter), Member 1 (Kidd Blood Group) |
| **SUSD4** | Sushi Domain Containing 4 |
| **SPATA9** | Spermatogenesis Associated 9 |
| **C14orf23** | Chromosome 14 Open Reading Frame 23 |
| **TMEM229B** | Transmembrane Protein 229B |
| **EFCAB1** | EF-Hand Calcium Binding Domain 1 |
| **GPR65** | G Protein-Coupled Receptor 65 |
|  | |
| *Common upregulated transcript after NOC silencing* | |
| **DPPA3** | Developmental Pluripotency Associated 3 |
|  | |
| *Common downregulated transcript after NOC silencing* | |
| **GUCA1B** | Guanylate Cyclase Activator 1B (Retina) |
