# Supplementary material for: Poly(A)-specific ribonuclease and Nocturnin in squamous cell lung cancer: prognostic value and impact on gene expression
Source: Mol Cancer. 2015 Nov 5;14:187. doi: 10.1186/s12943-015-0457-3 (PMC4635609; doi:10.1186/s12943-015-0457-3)
Supplement: Additional file 7: Table S6. — Functional Enrichment Analysis of genes with differentially increased expression after PARN silencing in both NCI-H520 and Hep2 cells. (DOCX 12 kb) [file 12943_2015_457_MOESM7_ESM.docx]

**Additional file 7: Table S6.** Functional Enrichment Analysis of genes with differentially increased expression after PARN silencing in both NCI-H520 and Hep2 cells.

| **Function** | **FDR** | **Coverage** |
| --- | --- | --- |
| **query genes** | **n/a** | **4 / 4** |
| *G-protein coupled receptor signaling pathway, coupled to cyclic nucleotide second messenger* | 4.48E-24 | 22 / 116 |
| *adenylate cyclase-modulating G-protein coupled receptor signaling pathway* | 4,03E-21 | 18 / 77 |
| *G-protein beta/gamma-subunit complex binding* | 4,17E-13 | 9 / 17 |
| *regulation of nucleotide metabolic process* | 3,37E-12 | 19 / 284 |
| *regulation of purine nucleotide metabolic process* | 3,37E-12 | 19 / 281 |
| *heterotrimeric G-protein complex* | 6,83E-12 | 9 / 23 |
| *extrinsic to internal side of plasma membrane* | 8,52E-12 | 10 / 36 |
| *extrinsic to plasma membrane* | 2,42E-11 | 11 / 57 |
| *regulation of cyclic nucleotide biosynthetic process* | 1.1E-9 | 11 / 80 |
| *extrinsic to membrane* | 1.14E-9 | 11 / 81 |
| *internal side of plasma membrane* | 1.16E-9 | 11 / 83 |
| *regulation of nucleotide biosynthetic process* | 1.16E-9 | 11 / 83 |
| *regulation of purine nucleotide biosynthetic process* | 1.16E-9 | 11 / 83 |
| *regulation of cyclic nucleotide metabolic process* | 1.23E-9 | 11 / 84 |
| *regulation of adenylate cyclase activity* | 3.84E-9 | 9 / 47 |
| *cyclic purine nucleotide metabolic process* | 3.84E-9 | 11 / 94 |
| *cyclic nucleotide biosynthetic process* | 4.07E-9 | 11 / 95 |
| *regulation of cyclase activity* | 7.48E-9 | 9 / 51 |
| *regulation of cAMP biosynthetic process* | 7.49E-9 | 10 / 74 |
| *regulation of cAMP metabolic process* | 8.17E-9 | 10 / 75 |
