# Supplementary material for: Poly(A)-specific ribonuclease and Nocturnin in squamous cell lung cancer: prognostic value and impact on gene expression
Source: Mol Cancer. 2015 Nov 5;14:187. doi: 10.1186/s12943-015-0457-3 (PMC4635609; doi:10.1186/s12943-015-0457-3)
Supplement: Additional file 8: Table S7. — Functional Enrichment Analysis of genes with differentially reduced expression after PARN silencing in both NCI-H520 and Hep2 cells. (DOCX 12 kb) [file 12943_2015_457_MOESM8_ESM.docx]

**Additional file 8: Table S7.** Functional Enrichment Analysis of genes with differentially reduced expression after PARN silencing in both NCI-H520 and Hep2 cells.

| **Function** | **FDR** | **Coverage** |
| --- | --- | --- |
| **query genes** | **n/a** | **14 / 14** |
| *glutamate receptor signaling pathway* | 5.21E-13 | 12 / 46 |
| *neuron part* | 9.06E-10 | 17 / 251 |
| *neuron projection* | 1.06E-9 | 16 / 219 |
| *ionotropic glutamate receptor complex* | 1.47E-9 | 8 / 22 |
| *regulation of excitatory postsynaptic membrane potential* | 9.99E-7 | 6 / 16 |
| *regulation of postsynaptic membrane potential* | 1.76E-6 | 6 / 18 |
| *protein localization to membrane* | 1.76E-6 | 9 / 77 |
| *positive regulation of excitatory postsynaptic membrane potential* | 6.61E-6 | 5 / 11 |
| *receptor complex* | 8.08E-6 | 10 / 128 |
| *dendritic spine* | 1.24E-5 | 7 / 45 |
| *neuron spine* | 1.24E-5 | 7 / 45 |
| *synapse* | 1.26E-5 | 10 / 138 |
| *positive regulation of membrane potential* | 1.74E-5 | 5 / 14 |
| *dendrite* | 2.53E-5 | 9 / 112 |
| *PDZ domain binding* | 2.59E-5 | 7 / 52 |
| *Wnt-activated receptor activity* | 7.73E-5 | 5 / 19 |
| *synaptic membrane* | 7.73E-5 | 6 / 37 |
| *cell projection part* | 1.03E-4 | 12 / 283 |
| *synapse part* | 1.23E-4 | 8 / 100 |
| *Wnt-protein binding* | 1.82E-4 | 5 / 23 |
