# Supplementary material for: Poly(A)-specific ribonuclease and Nocturnin in squamous cell lung cancer: prognostic value and impact on gene expression
Source: Mol Cancer. 2015 Nov 5;14:187. doi: 10.1186/s12943-015-0457-3 (PMC4635609; doi:10.1186/s12943-015-0457-3)
Supplement: Additional file 9: Table S8. — Functional Enrichment Analysis of genes with differentially reduced expression after NOC silencing in both NCI-H520 and Hep2 cells. (DOCX 11 kb) [file 12943_2015_457_MOESM9_ESM.docx]

**Additional file 9: Table S8.** Functional Enrichment Analysis of genes with differentially reduced expression after NOC silencing in both NCI-H520 and Hep2 cells.

| **Function** | **FDR** | **Coverage** |
| --- | --- | --- |
| **query genes** | **n/a** | **1 / 1** |
| *pigment granule* | 7.3E-4 | 3 / 16 |
| *melanosome* | 7.3E-4 | 3 / 16 |
| *regulation of G-protein coupled receptor protein signaling pathway* | 3.4E-2 | 3 / 63 |
| *secondary metabolic process* | 2.84E-1 | 2 / 22 |
| *aromatic amino acid family metabolic process* | 2.84E-1 | 2 / 23 |
| *pigment biosynthetic process* | 3.8E-1 | 2 / 29 |
| *copper ion binding* | 4.76E-1 | 2 / 35 |
| *pigment metabolic process* | 5.45E-1 | 2 / 40 |
